# Does prey encounter and nutrient content affect prey selection in wolf spiders inhabiting Bt cotton fields?

**DOI:** 10.1371/journal.pone.0210296

**Published:** 2019-01-10

**Authors:** Dalila Rendon, Phillip W. Taylor, Shawn M. Wilder, Mary E. A. Whitehouse

**Affiliations:** 1 CSIRO Agriculture and Food, Australian Cotton Research Institute, Narrabri NSW, Australia; 2 Department of Biological Sciences, Macquarie University, Sydney NSW, Australia; 3 Department of Integrative Biology, Oklahoma State University, Stillwater OK, United States of America; Agroscope, SWITZERLAND

## Abstract

Wolf spiders are abundant and voracious predators at the soil-plant interface in cotton crops. Among other prey, they attack late-instar larvae of the cotton bollworm *Helicoverpa* spp., an economically important pest. Consequently, wolf spiders in transgenic Bt cotton could provide significant biological control of Bt-resistant *Helicoverpa* larvae that descend to the soil to pupate. The predator-prey interactions between wolf spiders and *Helicoverpa* could, however, be constrained by the presence of alternative prey and intraguild predators. This study used laboratory enclosures to analyse the effect of alternative prey on predatory selection of the wolf spider *Tasmanicosa leuckartii* Thorell. The prey included another wolf spider *Hogna crispipes* Koch (potential intraguild predator), the ground cricket *Teleogryllus commodus* Walker (minor pest), and *Helicoverpa armigera* larvae (major pest). We tested if encounter rates, prey vulnerability, and prey nutritional content influenced the likelihood that a prey was attacked. In three-way food webs, *Tasmanicosa* encountered and attacked *Teleogryllus* and *Helicoverpa* in similar frequencies. However, in the presence of a competing intraguild predator and potential prey (*Hogna*) in a four-way food web, *Tasmanicosa* did not always attack *Teleogryllus* at first encounter, but still attacked *Helicoverpa* at each encounter. *Helicoverpa* (protein-poor) and *Hogna* (protein-rich) were consumed by *Tasmanicosa* in similar proportions, suggesting that *Tasmanicosa* might benefit from nutrient balance as an outcome of diverse prey in this food web. As *Teleogryllus* (protein rich) escapes quicker than *Helicoverpa* and *Hogna*, *Hogna* may be an easier protein-rich option than *Teleogryllus*. Field surveys showed that while *Teleogryllus* was the most common prey, wolf spiders feed on diverse insect taxa, as well as other spiders. That *Tasmanicosa* readily attacked *Helicoverpa* larvae in the presence of alternative prey is an encouraging result that supports the potential of *Tasmanicosa* predation to assist in the control of Bt-resistant *Helicoverpa* larvae and thereby inhibit the proliferation and spread of resistance.

## Introduction

Interactions between a predator and its prey rarely occur in isolation. Instead, predatory interactions usually occur within food webs that also involve primary producers, alternative prey, and other predators. When multiple prey are present, predation outcomes may be driven by prey availability (passive selection) or by predator-prey interactions upon prey encounter [1]. Variables influencing passive selection of prey include abundance, prey dispersion [2] and camouflage [3]. After encounter, predation outcomes may be determined by the prey’s ability to escape [4, 5], prey defences [5], prey size [6–8], predator hunger state [4, 9], or prey nutritional content [10, 11]. Predators may not attack prey that are difficult to catch and subdue, or impose risk of injury or toxicity. Optimal foraging theory combines these factors on predation outcomes, and posits that after predators encounter prey they should aim to maximize their energy intake [12] while minimizing the risks and energy spent in prey capture [13]. Understanding the mechanisms that mediate predation tendencies is crucial to predict the structure and function of food webs.

In agricultural food webs, generalist predators do not target pest prey exclusively, yet these predators are still an important component of integrated pest management [14]. Prey diversity can be essential to sustain generalist predator populations; when pest densities are low, predators may rely on alternative prey to sustain growth and survival, allowing the predator population to be maintained [15]. In some cases, a diet consisting of only single pest prey may be nutritionally deficient and can drastically reduce the survival of generalist predators [16]. As a result, foraging on diverse prey may enable predators to balance nutrient intake [17–19]. Alternative prey therefore may be essential for predators to thrive in agricultural systems. Because the presence of alternative prey may influence predator-prey interactions and food web dynamics, it is important to understand how the presence of alternative prey might affect the biological control of pest species.

Abundance of alternative prey may influence whether a generalist predator feeds on a particular pest predominantly or switches prey preference [20]. Interference of the biological control of pests due to the presence of alternative prey has been reported in several studies; for example, in the presence of Collembola (alternative prey), spiders may kill fewer pest aphids [15, 21, 22]. In other cases, generalist predators can still effectively suppress pest populations despite the availability of diverse prey. For example, the wolf spider *Hogna* sp. continues feeding on cucurbit pests, even when alternative prey are available [23]. Predators may also respond differently to different types of alternative prey. For instance, the wolf spider *Pardosa prativaga* consumes fewer aphids when vinegar flies (*Drosophila* spp.) are available, but does not change its predation rate on aphids when collembola are available [24]. The interactions between generalist predators, pest prey, and alternative prey are difficult to predict and vary among species and agricultural systems.

In cotton agroecosystems, biological control by predators can help suppress pest populations. For example, a predator complex of spiders, predacious bugs, lacewings, and predacious beetles are important for the biocontrol of whitefly *Bemisia tabaci*, and the mirid bug *Lygus* [25–27]. Similarly, the ladybeetle *Coccinella septempunctata* can be a key factor to supress populations of cotton aphids [28]. The bollworm, *Helicoverpa armigera* (Hübner) (Lepidoptera: Noctuidae; referred to hereafter as *Helicoverpa*) is a key pest of particular importance in cotton [29]. Genetically modified Bt-cotton produces toxins that destroy the gut lining of Lepidoptera, and was introduced to control the larvae of *Helicoverpa* spp. [30, 31]. However, the long term viability of Bt cotton is constantly threatened by the potential of *Helicoverpa* to develop resistance to Bt-toxins [32]. Bt-resistant larvae, like all *Helicoverpa*, descend from the cotton plants to pupate underground, and later emerge as moths that convey resistance traits to the next generation. After foraging on cotton, these larvae are exposed to predators on the plant-soil interface. By killing larvae of *Helicoverpa* as they descend to pupate, or later as they emerge as moths, ground predators present in cotton crops can inhibit the proliferation of Bt-resistant genes in *Helicoverpa* populations, as it has been shown with other Lepidoptera pests [33]. Because Bt cotton fields require fewer insecticide spray applications than conventional cotton, they can harbour a diverse and sizeable arthropod community [34, 35]. This arthropod diversity may serve as alternative prey to sustain predator populations in Bt cotton fields.

In the present study, we examine the predator-prey interactions of four arthropods commonly found on the soil surface of cotton fields in New South Wales, Australia. Wolf spiders (Araneae: Lycosidae) are commonly classified as generalist predators, and will readily accept crickets, Lepidoptera larvae, and other wolf spiders as prey [36]. The wolf spiders *Tasmanicosa leuckartii* (Thorell) and *Hogna crispipes* Koch; (referred to hereafter as *Tasmanicosa* and *Hogna*) are abundant in cotton fields, with *Hogna* representing approximately 35% and *Tasmanicosa* representing approximately 12% of the wolf spider community [37]. Both spider species attack late-instar *Helicoverpa* larvae as they descend from the plant to pupate in the soil, and emerging adults on the soil [38, 39]. The ground cricket *Teleogryllus commodus* Walker (Orthoptera: Gryllidae; referred to hereafter as *Teleogryllus*) is also commonly found in cotton fields, and has been reported to be an occasional pest in cotton when they are abundant. Adults and late-instar nymphs can be early-season pests, as they feed on the leaves and stems of cotton seedlings [40]. This ground food web includes therefore two abundant predators in Bt cotton fields (*Tasmanicosa* and *Hogna*), an abundant but economically minor cotton pest (*Teleogryllus*), and a less abundant, yet economically important cotton pest (*Helicoverpa*).

Here, we evaluate how interactions between *Tasmanicosa*, *Hogna*, *Teleogryllus* and *Helicoverpa* influence predation outcomes. First, we test the hull hypothesis that prey attack probability is independent of prey first encounter. If *Tasmanicosa* is a generalist predator in this food web with no prey preference for *Hogna*, *Teleogryllus* or *Helicoverpa*, we then expect that the first prey encountered will be the first prey attacked. Additionally, we investigate whether prey protein and lipid contents of *Hogna*, *Teleogryllus*, and *Helicoverpa* influence prey selection in *Tasmanicosa*.

## Materials and methods

### Collection of spiders and *Teleogryllus*

This study was carried out at the Australian Cotton Research Institute (ACRI; 33°S, 149°E), near Narrabri, New South Wales, Australia. Adult males, females and late-instar juveniles of the wolf spiders *Tasmanicosa* (cephalothorax width = 7.1 ± 1.2 mm; mean ± SD); and *Hogna* (cephalothorax width 5.7 ± 1.1 mm; mean ± SD) were collected in and around Bt-cotton fields after sunset from December until March 2014. Because *Teleogryllus* nymphs shelter in soil cracks and are difficult to collect from cotton fields, *Teleogryllus* nymphs (body length = 12.8 ± 2.4 mm, mean ± SD) were collected around the buildings at ACRI. Spiders and crickets were found by visual search using a headlamp (Petzl Tikka, 140 lumens), and collected manually in clear 70 ml cylindrical plastic containers. After collecting, all spiders and crickets were brought to the laboratory. Spider cephalothorax width and cricket body length were measured to the nearest 0.01 cm using a manual caliper (resolution 0.01 cm). Each spider and cricket was weighed to the nearest 0.01 g using a digital scale (Sartorius model A200S, Gottingen, Germany).

Collected spiders were housed in clear plastic containers (220 mm height x 228 mm length x 228 mm width, 8.5 L, Décor Tellfresh superstorer, NSW, Australia; referred to hereafter as ‘spider container’) containing 2 L of moist soil, and were kept in a controlled environment room (24.4 ± 0.5 ^o^C, mean ± SD) with a L14:D10 photoperiod. Each container had a retreat in each of two opposite corners comprising a hole in the soil approximately 2 cm deep and 1 cm diameter. The retreat was partially covered by a 3 x 3 cm sheet cut from the bark of a ‘Paper Bark tree’ (*Melaleuca* sp.*)*. All spiders were isolated by placing an opaque PVC pipe (100 mm diameter x 150 mm height) over each spider’s retreat until experiments started. *Teleogryllus* were kept in the same 70mL plastic clear containers used for collection. Each spider and *Teleogryllus* were kept in the laboratory in their respective containers for ~ 20 h before being used in food web experiments, and during this period no food or water were supplied. All alive spiders and crickets were released in the fields after trials.

### *Helicoverpa* predation

This experiment assessed whether *Tasmanicosa* kills both *Helicoverpa* larvae reared in artificial diet (used in food web experiments) and larvae reared in cotton plants (field scenario). Larvae of *Helicoverpa armigera* were supplied by the Commonwealth Scientific and Industrial Research Organization (CSIRO) Agriculture Flagship Bt Resistance Monitoring Group. Larvae were reared in individual wells in trays with soy and agar diet [41, 42]; referred to as ‘diet-reared’ larvae), and kept in a controlled environment room (24.4 ± 0.5 ^o^C, mean ± SD) with a L14:D10 photoperiod. A separate group of larvae were reared from neonates on plant material (referred to as ‘plant-reared’ larvae). Plant-reared larvae were placed in individual wells in trays containing a mixture of cotton leaves, flowers, and squares (Sicot 71 conventional, non-Bt, RRF). Larvae were transferred into new trays with fresh plant material every two days. Both diet-reared and plant-reared larvae were maintained until they reached 5^th^ instar (the last instar on the plant before burrowing underground to pupate) and were then weighed to the nearest 0.01 g (body weight = 0.40 ± 0.07 g, mean ± SD) using a digital scale (Sartorius model A200S).

To assess whether spiders kill both plant-reared and diet-reared larvae, one *Tasmanicosa* was paired simultaneously with one diet-reared 5^th^ instar larva and one plant-reared 5th instar larva (N = 10). To distinguish larvae, both were marked with different colour dyes (HCA Colours, Kingsgrove NSW, Australia; VM311 Pink and VM315 Orange), alternating colours in different trials (so that five plant-reared larvae and five diet-reared larvae had pink dye, and five of each had orange dye). Larval mortality from predation was recorded after 24 h.

### First encounter and first attack in three-way and four-way food webs

To determine the effect of alternative prey and intraguild predators on the first prey attacked by *Tasmanicosa*, three-way (*Helicoverpa–Teleogryllus–Tasmanicosa*; N = 27) and four-way (*Helicoverpa–Teleogryllus–Hogna–Tasmanicosa*; N = 23) food webs were set up in which animals were present together in the same spider container. All food web experiments were carried out in the same controlled environment room described above (see *Collection of spiders and* Teleogryllus). Due to the high mortality and low sample size of plant-reared *Helicoverpa* larvae, all food web experiments were carried out using diet-reared *Helicoverpa* larvae. Approximately 30 minutes after the dark phase commenced in the controlled environment room, one 5^th^ instar *Helicoverpa* larva and one *Teleogryllus* nymph were placed inside a container housing either (1) one *Tasmanicosa;* or (2) one *Tasmanicosa* and one *Hogna*. Because we were interested in analysing the predatory behaviour of *Tasmanicosa* as a focal predator, *Hogna* was always smaller than *Tasmanicosa* to avoid bi-directional intraguild predation. PVC pipes were immediately removed to allow spiders to explore and hunt. Continuous video recording began immediately after *Helicoverpa* and *Teleogryllus* were released, and ended 24 h later. The recording system comprised a 1/3” CCD monochromatic infra-red camera (CCS- Sony Go Video, Sony) with a 4 mm C mount lens positioned above each container, which recorded to a 2TB DVR4-100 hard drive recorder. One infrared illuminator (IR-covert, 940 nm) was placed 10 cm to the side of each spider container. To improve video contrast, each animal was dusted with fluorescent dye (HCA Colours Australia, Kingsgrove, NSW; VM311 Pink for *Helicoverpa* larvae and *Teleogryllus*, VM315 for *Hogna*, and VM317 Yellow for *Tasmanicosa*). A previous study showed that dust dyes did not affect predatory behaviour [39]. For each trial, we recorded which prey item was first encountered (physical contact between predator and prey) and first attacked by *Tasmanicosa* (the spider lunging towards the prey).

### Nutritional analysis

To determine the relationship between prey protein and lipid content, and predation outcomes in food webs, a protein and lipid analysis was carried out in *Tasmanicosa*, *Hogna*, *Teleogryllus* and *Helicoverpa*. Because spiders were caught from the field 24 h before experiments and were not offered prey before trials, we made the assumption that spiders were responding to the *Helicoverpa* offered to them as if the prey were sourced from the field, and would not respond differently to a diet-reared *Helicoverpa*. Therefore, we assumed that any link between predation outcomes and nutrient content would be based on the typical protein and lipid contents of prey in the field. Juvenile *Tasmanicosa* (N = 8), juvenile *Hogna* (N = 12), field-collected *Teleogryllus* (N = 12), diet-reared *Helicoverpa* (N = 21), and plant-reared *Helicoverpa* (N = 12) were collected for nutritional analysis. *Tasmanicosa*, *Hogna* and *Teleogryllus* were collected from the same fields described above, and immediately frozen at -20°C for nutritional analysis. Diet-reared and plant-reared *Helicoverpa* larvae were reared as described above, and frozen at -20°C once they reached 5^th^ instar.

Protein and lipid analyses were performed following protocols previously tested in arthropods [43, 44]. After freezing, each arthropod was dried in an oven at 60 ^o^C for 48 h before lipid and protein analysis. Lipid content was measured gravimetrically by submerging each dried arthropod in chloroform for 24 h, discarding the chloroform, repeating for another 24 h, and then drying again. The lipid content was estimated by taking the difference in the dry weight of samples before and after soaking them in chloroform. Protein was extracted from ground sub-samples (3–5 mg) using 0.1 M NaOH and heat (90°C for 30 minutes) after which samples were centrifuged and the supernatant was collected for analysis. Protein content was then measured using the Bradford Assay modified for use in 96 well microplates following manufacturer’s instructions (protein assay kit #500–001, Bio-Rad, Hercules CA, USA). We analysed each sample in triplicate and all samples were run together on the same plate reader (Biotek EL808, Vinooski, VT, USA) with a calibration curve created using a protein standard (bovine gamma globulin, Bio-Rad #500–001).

### Wolf spider prey in cotton fields

A field survey was carried out to determine common prey of wolf spiders in a mixed cotton field. This survey was carried out between 26 February and 7 March 2015, between the ‘peak flower’ and ‘open boll’ stage of cotton growth [45], which is the period when adult wolf spiders are most abundant in cotton fields at ACRI [37]. Surveys were carried out on dry nights, as wolf spiders remain in their burrows during rain (personal observation). Visual surveys took place around the edge of a triangular shaped cotton field 240m wide (= 240 rows: 1 cotton row/ m) with row lengths ranging from 60 m to 160 m. This mixed cotton field comprised Bt, non-Bt, and pigeon pea refuge plantings. Even though wolf spiders are abundant at this stage of the season, the plant canopy already covered the plot rows, precluding visual search within the crop. Therefore, only the 3 m around the edges of the plant rows of the field were surveyed for wolf spiders for 2hrs after sunset. Nocturnal wolf spiders hunt more actively immediately after sunset, and feeding tends to decline over the following hours [46]. During surveys, for every spider with a cephalothorax width greater than approximately 3.5mm we recorded its species, and whether it was holding a prey in its chelicerae. Because juvenile *Tasmanicosa* and *Hogna* can have similar cephalothorax patterns and are difficult to differentiate in the field, all juvenile spiders without a characteristic adult or subadult pattern were classified as “other Lycosidae”. All spiders holding a prey were captured and taken to the laboratory for prey identification.

### Statistical analysis

A test of independence with *post-hoc* adjusted residual z values was used to determine if frequencies of first attack were independent of first encounter for *Tasmanicosa* towards *Hogna*, *Teleogryllus* and *Helicoverpa*. Percent protein and percent lipid in arthropods were analysed for normality using a Shapiro-Wilk test, log-transformed where necessary and three extreme outlier values were removed (based on biologically unrealistic protein values that could only reflect errors) to meet the assumptions of parametric testing. A multivariate analysis of variance (MANOVA) was used to test for differences in percent lipid and protein between diet-reared *Helicoverpa*, plant-reared *Helicoverpa*, field-collected *Teleogryllus*, and field- collected *Hogna* and *Tasmanicosa*, using dry body mass as a covariate. Post-hoc least significant differences (LSD) were carried out to determine differences in percent lipid and percent protein separately between each arthropod. All statistical analyses were carried out using SPSS [47].

### Ethics statements

Animal research: no humans, vertebrates, or cephalodods were used in this study

Field studies: Prey field observation and collection was done at the Australian Cotton Research Institute; no permits were required for CSIRO staff (M. Whitehouse and D. Rendon).

## Results

### *Helicoverpa* predation

At the end of *Helicoverpa* predation trials, all spiders had killed both the plant-reared and the diet-reared larvae. In six out of ten trials, *Tasmanicosa* killed plant-reared *Helicoverpa* first (regardless of coloured dye dust), no larvae were rejected, and both *Helicoverpa* larvae were completely consumed.

### First encounter and first attack in three-way and four-way food webs

**Fig 1:**
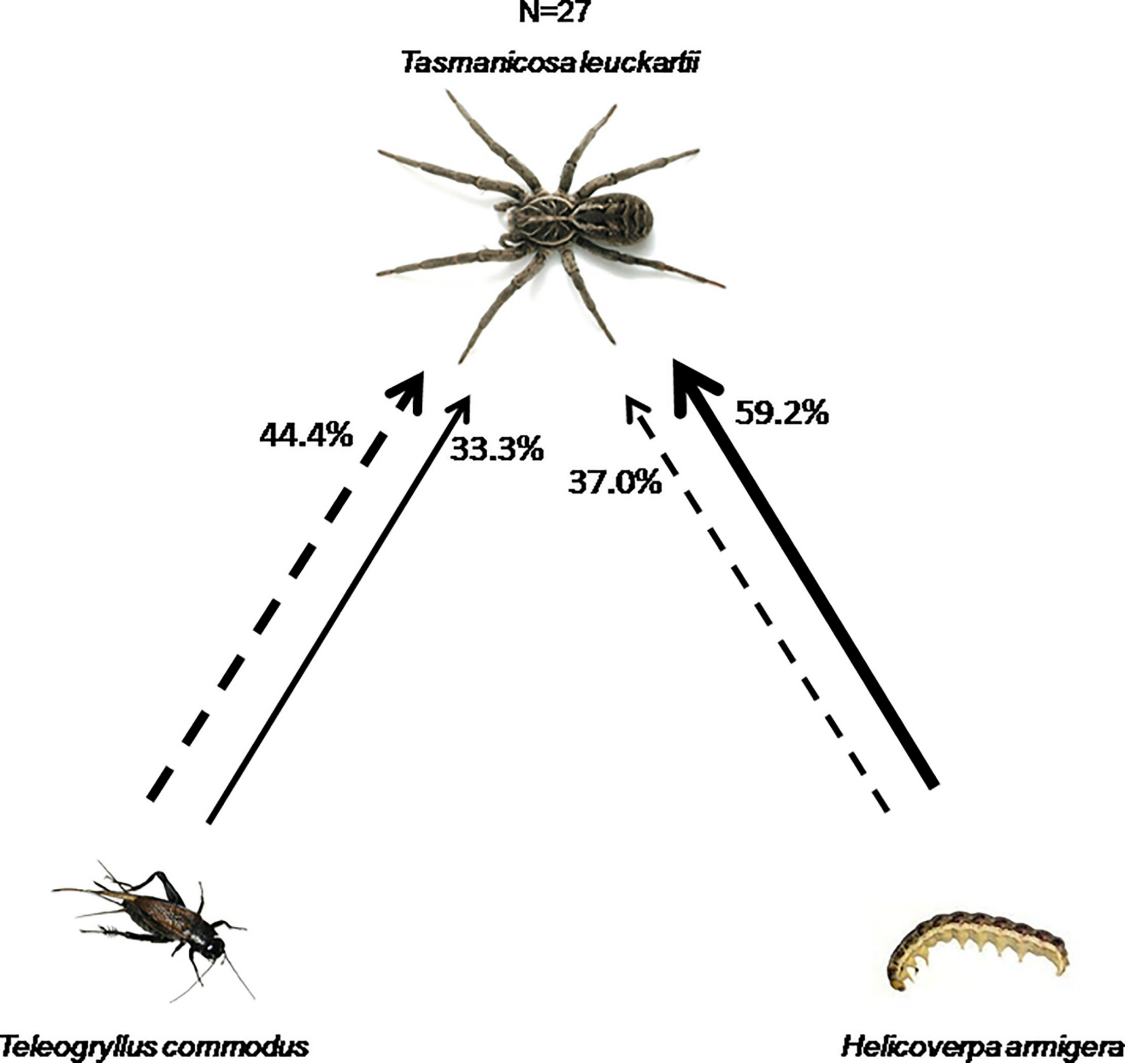
Percentages of first encounter (dashed arrows) and first attack (solid arrows) of *Tasmanicosa* in a three-way videorecorded food web arena. Direction of arrows point to the predator that made the encounter/attack, thicker arrows represent higher proportions. Percentages indicate cases in which prey was encountered first (out of n = 27), or attacked first (out of n = 27).

In three-way food webs, there were no differences in the proportions of first encounters between *Tasmanicosa* and *Teleogryllus* or *Helicoverpa* (Pearson χ^2^ = 0.30, df = 1, p = 0.58; Fig 1). Frequency of first attack was not random, and was associated with frequency of first encounter (χ^2^ = 11.81, df = 1, p < 0.01; Fig 1); *Tasmanicosa* attacked more frequently the first prey encountered, regardless of whether it was *Teleogryllus* or *Helicoverpa*. After being first encountered, 90% of *Helicoverpa* (N = 10) and 75% of *Teleogryllus* (N = 12) were immediately attacked by *Tasmanicosa*.

**Fig 2:**
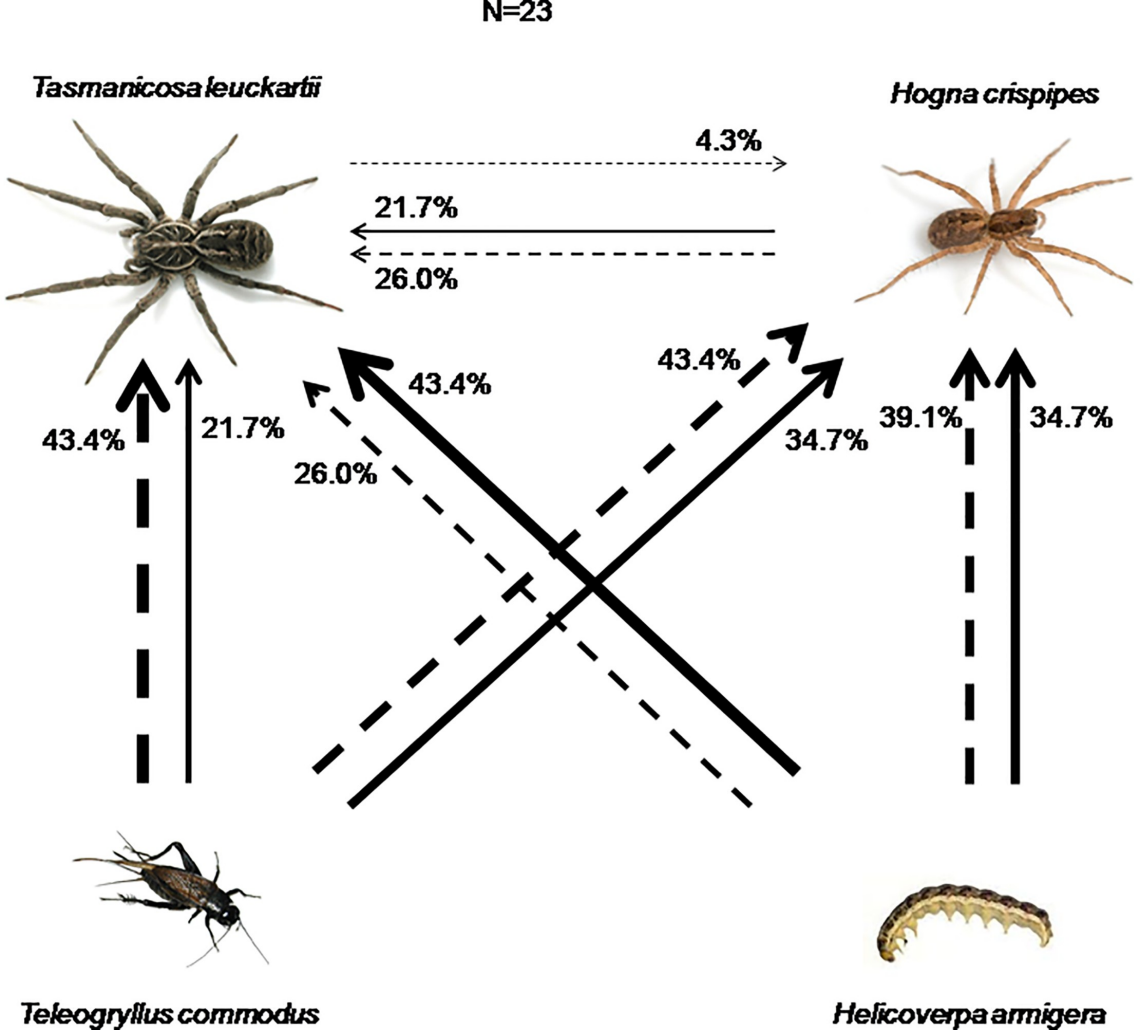
Percentages of first encounter (dashed arrows) and first attack (solid arrows) of *Tasmanicosa* and *Hogna* in a four-way videorecorded food web arena. Direction of arrows point to the predator that made the encounter/attack, thicker arrows represent higher proportions. Percentages indicate cases in which prey was encountered first (out of n = 23), or attacked first (out of n = 23).

Similarly, in four-way food webs, there were no differences in the proportions of first encounters between *Tasmanicosa* and *Hogna*, *Teleogryllus*, or *Helicoverpa* (Pearson χ^2^ = 2.40, df = 2, p = 0.30, cases in contingency table = 40; Fig 2). *Hogna* attacked and killed *Teleogryllus* and *Helicoverpa* each 34.7% of the time, but this did not affect the proportion of first encounter of *Telegogryllus* and *Helicoverpa* by *Tasmanicosa*. Frequency of *Tasmanicosa* first attack was not random, and was associated with frequency of first encounter (χ^2^ = 11.35, df = 4, p = 0.023; Fig 2). Post-hoc tests revealed that *Helicoverpa* and *Hogna* were often attacked first by *Tasmanicosa*, even when they were not the first prey encountered (all z < 1.96), while *Teleogryllus* was not always attacked when it was encountered first (adjusted residual z = 2.9).

No prey escaped a spider attack, and all attacks resulted in the spider completely consuming the prey. When attacked by a spider, *Helicoverpa* displayed behaviours such as biting, bobbing its body from side to side, ejecting faeces and regurgitating. Larger *Helicoverpa* larvae sometimes lifted an attacking spider off the ground. None of these *Helicoverpa* behaviours were life threatening for a spider, and no spider died from attacks on *Helicoverpa*, or retreated after initiating attack. When in contact with or being seized by a spider, *Teleogryllus* exhibited behaviours such as kicking and head-butting. No spiders died from injuries inflicted by *Teleogryllus*. *Teleogryllus* usually jumped away when coming into contact with a spider, or even when a spider moved within a few centimetres. *Hogna* usually ran away after detecting an approaching *Tasmanicosa*, but never counter-attacked or bit, and were quickly seized by larger spiders. No *Tasmanicosa* died from attacking *Hogna*.

### Lipid and protein content

**Fig 3:**
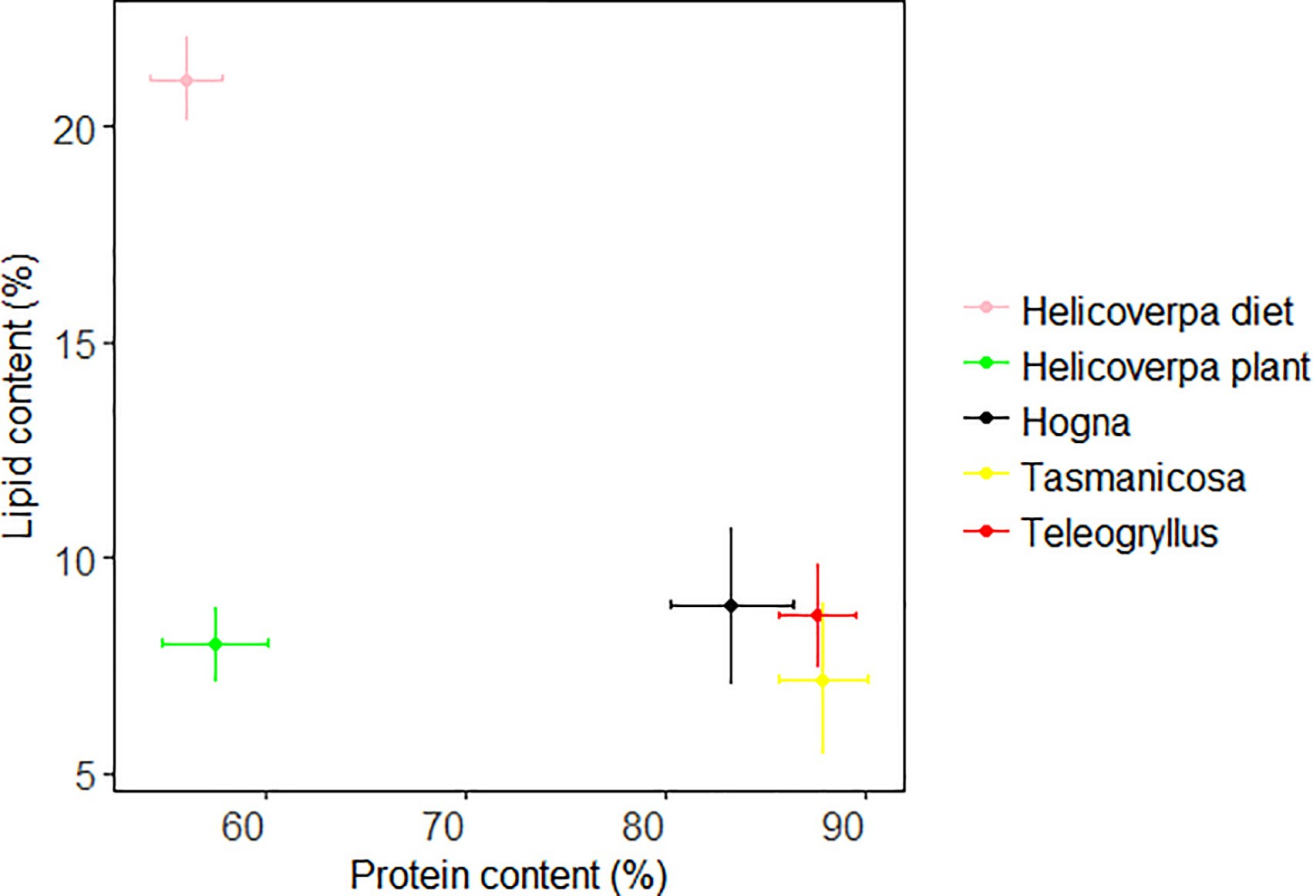
Percent body content of protein and lipid in four arthropods (mean and SD), caught near cotton fields, reared on cotton plant material, or reared on soy and agar diet.

Comparing protein and lipid contents of all arthropods, we found that protein content was lowest in plant-reared *Helicoverpa*, and was similar among field-collected *Teleogryllus*, *Hogna* and *Tasmanicosa*. (Wilks Lambda = 0.07, F = 32.69, df = 3, 39, p<0.01, post-hoc LSD; Fig 3). Lipid contents were not different among plant-reared *Helicoverpa*, and field-collected *Teleogryllus*, *Hogna* and *Tasmanicosa* (Wilks Lambda = 0.07, F = 1.02, df = 3, 39, p = 0.39).

Diet changed lipid but not protein contents in *Helicoverpa*; diet-reared *Helicoverpa* had higher lipid content than plant-reared *Helicoverpa* (Wilks’ Lambda = 0.19, F = 91.13, df = 1, 31, p < 0.01; Fig 3), whereas diet-reared *Helicoverpa* had similar protein content as plant-reared *Helicoverpa* (Wilks’ Lambda = 0.19, F = 0.23, df = 1, 31, p = 0.63).

### Wolf spider prey in cotton fields

A total of 597 wolf spiders were observed in the fallow 3 m beyond the plant rows of the cotton fields, and 18 spiders were recorded holding prey (3.0%; Table 1). Some prey items (including Lepidoptera) could not be identified to species because they had been masticated by the spider. The most common prey was the ground cricket *Teleogryllus commodus* (33% of all prey observed). Two spiders were observed consuming another spider, confirming the occurrence of intraguild predation in the field.

**Table 1:** Wolf spider prey observed in visual surveys around edges of cotton fields.

	*Tasmanicosa leuckartii* (N = 114)	*Hogna crispipes* (N = 90)	Other *Lycosidae* (N = 393)
**Orthoptera**			
Gryllidae: *Teleogryllus commodus*	**0**	**1**	**5**
**Dermaptera**			
Labiduridae: *Labidura truncate*	**1**	**0**	**2**
**Lepidoptera** moth	**0**	**0**	**2**
**Lepidoptera** caterpillar	**0**	**1**	**0**
**Lycosidae**	**0**	**0**	**2**
**Coleoptera**			
Scarabeidae: *Mimadoretus sp*.	**0**	**0**	**1**
**Hymenoptera**			
Formicidae: *Iridiomyrmex* sp.	**0**	**0**	**1**
**Unknown**	**0**	**0**	**2**

## Discussion

In the present study, we observed that in the presence of alternative prey, *Helicoverpa* was still targeted as prey by *Tasmanicosa*. Predation outcomes can result from a combination of prey driven (passive selection) or predator driven (active selection) variables.

Wolf spiders are considered generalist predators that exhibit little prey selectivity, tending to feed according to availability. Wolf spiders respond principally to prey movement [48], and have been assumed to attack whenever a moving suitable prey is detected and comes within reach [49]. From three-way and four-way food web trials, we observed that *Tasmanicosa* encountered all prey at a similar rate. However, in four-way webs, *Tasmanicosa* did not always attack *Teleogryllus* at first encounter. This mismatch may be influenced by both the presence of an intraguild predator (*Hogna*), and the prey’s defence mechanisms. Prey encounters [50] or prey abundance [22] are not always the decisive factor for predation tendencies in hunting spiders. Other intrinsic characteristics of available prey should be taken into account to understand the factors that drive frequency of predation, such as prey’s defences or ability to escape.

Because antipredatory behaviour and prey choice are not mutually exclusive in determining predation outcomes, disentangling their confounding effects is not straightforward. In some cases, passive selection mechanisms underlie what might seem to be a predator’s active choice. For example, predatory midge larvae (*Chaoborus*) appear to select prey that are medium-sized, but this size selection is in fact confounded by the rate of prey encounter and capture success, thereby indicating a combination of passive prey selection and active predator choice [2]. Other studies support the hypothesis of active prey choice regardless of passive selection variables. For example, predatory mirids selected two-spotted mites over phytoseiid mites regardless of how easily they are found and captured, suggesting that mirids actively choose prey based on nutritional benefits [5]. In four-way food webs, *Tasmanicosa* did not always attack *Teleogryllus* after first encounter. This suggests that predation outcomes were not always determined by rate of encounter, and that prey vulnerability and the predator’s response to such vulnerability can mediate the food web connections between *Tasmanicosa*, *Hogna*, *Teleogryllus* and *Helicoverpa*.

Potential risks and costs associated with attacking prey can be an important predictor of predatory decisions. Risks could involve physical injury or death due to counter attack, while costs involve energetic expenditure for subduing prey such as chasing and restraining. In the present trials, *Helicoverpa* and *Teleogryllus* exhibited defence behaviours against *Tasmanicosa*. Since *Tasmanicosa* was never physically injured while attempting to subdue *Teleogryllus* and *Helicoverpa*, it could be assumed that under the circumstances and prey sizes used for this study, *Teleogryllus* and *Helicoverpa* pose a similarly low risk to *Tasmanicosa*. Additionally, the body-bobbing behaviour of *Helicoverpa* might intensify the spider’s attack behaviour as a visual stimulus [51]. Being venomous predators themselves, spiders should pose the highest risk for predation. However, in four-way food webs *Hogna* did not counter-attack as a defence mechanism, and was usually attacked after first encounter. In fact, in the presence of *Hogna*, *Tasmanicosa* did not always attack *Teleogryllus* at first encounter, suggesting that *Tasmanicosa* might benefit from instead consuming a competitor intraguild predator [52].

The ability of prey to escape and how a predator responds to escaping prey likely underlie predation outcomes. Compared to *Helicoverpa* and *Hogna*, *Teleogryllus* is a very mobile prey. After encounter, *Tasmanicosa* might not even have the chance to attempt an attack if *Teleogryllus* quickly jumps away. Dangles et al. (2006) found that wood crickets (*Nemobius*) could detect a wolf spider (*Pardosa*) 5mm away and still escape [53]. Additionally, attack success was correlated to prey distance and attack velocity, but wolf spiders did not modify their attack velocity depending on prey distance. Some crickets can also detect chemical cues from wolf spiders and modify their behaviour to avoid predation [54], and therefore crickets might not need to see or touch the spider to escape. From a predator’s perspective, *Tasmanicosa* had a 100% success rate at killing *Teleogryllus* after attacking in an enclosed container, but *Teleogryllus* might be better at escaping attacks in field conditions. If *Tasmanicosa* had experienced failed attacks towards *Teleogryllus* in the field, it is possible that spiders chose not to attack *Teleogryllus* immediately after encounter to avoid wasting energy in failed predation attempts. Thus, both *Teleogryllus’* ability to escape and *Tasmanicosa* hesitance to attack a quick prey can mediate predation outcomes in this food web. In the presence of slower prey such as *Helicoverpa* and *Hogna*, *Teleogryllus* is relatively costly and difficult to pursue, and may therefore represent a less vulnerable prey to *Tasmanicosa* than *Helicoverpa* and *Hogna*. This may reduce *Tasmanicosa’s* tendency to pursue *Teleogryllus*, particularly as *Hogna* had a similar protein and fat content to *Teleogryllus*.

Spider nutrition also has the potential to influence prey selection, yet few studies have explored the links between spider nutrition and food webs [55]. Nutrient intake by predators is important to regulate development, health, and reproduction [56], therefore prey selectivity in hunting spiders may be mediated by nutrient optimization and toxin aversion [57]. Some studies have argued that predatory arthropods are limited by nitrogen intake which is necessary for building proteins [17, 58]. From this perspective, spiders might benefit more by consuming prey with higher nitrogen content, such as other predators or omnivores [59], as nitrogen content enhances growth rates and survival in spiders [60], and herbivores tend to have lower protein content [61]. However, this nitrogen-limitation view has been challenged [62], arguing that the predator’s life stage, the way the predator differentially extracts nutrients, and the value of other macronutrients such as lipids and carbohydrates have a stronger effect on nutrient-mediated arthropod food webs. In our study, we did not control for hunger state, as spiders were not offered any prey before trials. Hunger state can also influence prey selection outcomes, and future studies should explore how spiders’ starvation levels or body nutrient contents directly relate to predation outcomes.

Even though food web trials were carried out using diet-reared *Helicoverpa*, our first experiment with *Helicoverpa* predation suggests that *Tasmanicosa* did not discriminate between diet-reared or plant-reared *Helicoverpa*. *Tasmanicosa* did not reject protein-poor prey (*Helicoverpa)* in the presence of protein-rich prey (*Teleogryllus* and *Hogna)*. By having lower protein and similar lipid contents as *Hogna*, *Helicoverpa* might not seem to provide a nutritional advantage to *Tasmanicosa*, especially considering that the gut of *Helicoverpa* in the field is likely to have a high content of plant cellulose, a carbohydrate indigestible to the spider. Welches et al. (2016) found that spiders often choose to pursue low-quality aphid prey, even when high-quality alternative prey are available [63]. Furthermore, there is evidence that wolf spiders (*Lycosa helluo*) develop quicker and survive longer on a diet of mixed arthropods [64]. Spiders might benefit from varied proportions of different amino acids and essential micronutrients rather than just bulk protein [65]. Greenstone (1979) found that the wolf spider *Pardosa ramulosa* preys on three different species of flies (Diptera: *Ephydra*, *Trichocorixa* and *Aedes*) in quantities enabling the proportions of essential amino-acids reflect those present in the spider’s haemolypmph [66]. *Helicoverpa* contains essential amino acids and digestible carbohydrates [67] which can contribute to a balanced nutrient intake. Other spiders can represent as much as 38% of a wolf spider’s mixed diet (for a review, see [52]), yet a diet consisting solely of conspecifics can be detrimental to spider development. For example, Oelbermann and Scheu (2002) found that lycosid spiderlings fed only spiders died sooner than spiderlings fed fruit flies or aphids, suggesting that conspecifics, despite their high protein content, still lack essential nutrients for development and survival [68]. Hence, similar predation rates on *Helicoverpa* and *Hogna* could represent diet mixing by *Tasmanicosa* to diversify their diet.

Field surveys showed that wolf spiders do feed on a diverse diet, composed of various insect orders. In the field, *Teleogryllus* was the most common prey. In this study, we did not quantify the abundance of each insect taxa, therefore we cannot determine if the frequency of prey attacked was linked to prey encounter in the field. Other studies suggest that spiders diversify their diet independently of prey availability, as some prey items are overrepresented in the diet relative to their abundance [69]. Our spider enclosure studies suggest that wolf spiders would likely feed on *Helicoverpa*, even if *Teleogryllus* is more common, and *Helicoverpa* is scarcer in cotton fields. The relationship between prey availability and prey selection in this cotton food web warrants further study.

From a pest control perspective, the results of the present study show that *Tasmanicosa* still kills *Helicoverpa* even when other common prey are available. In an agricultural landscape dominated by Bt-cotton, *Helicoverpa* larvae are less commonly encountered than are other wolf spiders or *Teleogryllus*. Predators often become more adept at killing and handling common prey, which may lead to development of a preference over unfamiliar prey [20]. However, our results indicate that *Tasmanicosa* would likely kill upon encounter any rare *Helicoverpa* larva that has succeeded in developing on Bt cotton in the same field as many other more common prey. The presence of various alternative prey ensures that there is an abundant population of spiders that can contribute to eliminate any Bt survivor larva. The results of this study thereby support the value of *Tasmanicosa* as an effective predator that can contribute to the control of Bt-resistance in *Helicoverpa*.
